# Clostridium Difficile and COVID-19: General Data, Ribotype, Clinical Form, Treatment-Our Experience from the Largest Infectious Diseases Hospital in Western Romania

**DOI:** 10.3390/medicina57101099

**Published:** 2021-10-13

**Authors:** Adelina Raluca Marinescu, Ruxandra Laza, Virgil Filaret Musta, Talida Georgiana Cut, Raluca Dumache, Anca Tudor, Mirela Porosnicu, Voichita Elena Lazureanu, Monica Licker

**Affiliations:** 1 Department XIII, Discipline of Infectious Diseases, University of Medicine and Pharmacy “Victor Babes”, 300041 Timisoara, Romania; marinescu_adelina24@yahoo.com (A.R.M.); ruxi_martincu@yahoo.com (R.L.); musta.virgil@umft.ro (V.F.M.); lazureanu.voichita@umft.ro (V.E.L.); 2 Clinical Hospital of Infectious Diseases and Pneumophtisiology “Doctor Victor Babes” Timisoara, 300310 Timisoara, Romania; mireitm84@yahoo.com; 3 Doctoral School, University of Medicine and Pharmacy “Victor Babes”, 300041 Timisoara, Romania; 4 Center for Ethics in Human Genetic Identifications, University of Medicine and Pharmacy “Victor Babes”, 300041 Timisoara, Romania; raluca.dumache@umft.ro; 5 Department VIII, Discipline of Forensic Medicine, Bioethics, Deontology and Medical Law, University of Medicine and Pharmacy “Victor Babes”, 300041 Timisoara, Romania; 6 Department III, Discipline of Informatics and Medical Biostatistics, University of Medicine and Pharmacy “Victor Babes”, 300041 Timisoara, Romania; atudor@umft.ro; 7 Department XIV, Discipline of Microbiology, University of Medicine and Pharmacy “Victor Babes”, 300041 Timisoara, Romania; licker.monica@umft.ro; 8 Multidisciplinary Research Centre on Antimicrobial Resistance, University of Medicine and Pharmacy “Victor Babes”, 300041 Timisoara, Romania

**Keywords:** clostridium difficile infection (CDI), COVID-19 pandemic, risk factors, outcome, ribotype, antibiotic usage

## Abstract

*Background and Objectives*: In Coronavirus Disease 2019 (COVID-19), which is caused by the infection with severe acute respiratory syndrome coronavirus 2 (SARS-CoV-2), the clinical manifestations are primarily related to the pulmonary system. Under 10% of cases also develop gastrointestinal events such as diarrhea, nausea, vomiting and abdominal pain. *Materials and Methods*: We conducted an observational, retrospective study in the Infectious Diseases Clinic of “Victor Babes” Hospital, Timis County, in order to assess the incidence, outcome and risk factors for clostridium difficile infection (CDI) in COVID-19 patients. *Results*: Out of 2065 COVID-19 cases, hospitalized between 1st September 2020 and 30th April 2021, 40 cases of CDI were identified with 32 cases of hospital-onset of CDI and eight cases of community-onset and healthcare-associated CDI. By randomization, polymerase chain reaction ribotyping of Clostridium Difficile was performed in six cases. All the randomized cases tested positive for ribotype 027. The percentage of cases recovered with complications at discharge was higher among COVID-19 patients and CDI (*p* = 0.001). The in-hospital stay, 36 days versus 28 days, was longer among COVID-19 patients and CDI (*p* = 0.01). The presence of previous hospitalization (*p* = 0.004) and administration of antibiotics during the hospital stay, increased the risk of CDI among COVID-19 patients. The mean adjusted CCI at admission was lower among controls (*p* = 0.01). In two cases, exitus was strictly CDI-related, with one case positive for 027 ribotype. *Conclusions*: CDI has complicated the outcome of COVID-19 patients, especially for those with comorbidities or previously exposed to the healthcare system. In the face of the COVID-19 pandemic and the widespread, extensive use of antibiotics, clinicians should remain vigilant for possible CDI and SARS-CoV-2 co-infection.

## 1. Introduction

Until 10th of May 2021, the COVID-19 pandemic, the largest modern epidemiologic event after the Great Spanish Flu of 1918 [1], reported more than 157 million confirmed cases with a total of 3,288,455 deaths [2].

In Romania, between 26 February 2020, when the first case was reported, and the end of 2020, a total of 632,263 cases and 15,767 COVID-19 related deaths have been registered. By 10th of May 2021, the total of COVID-19 cases reached 1,066,731 with 29,034 recorded deaths. In Timis county, official records stated a total number of 25,916 COVID-19 cases in 2020 and a total of 53,822 cases until 10th of May 2021 [3].

COVID-19, caused by the infection with SARS-CoV-2, a new RNA zoonotic virus of the *Coronaviridae* Family [4,5], is typically represented by pulmonary involvement such as bilateral pneumonia, consisting of extensive interstitial and alveolar inflammatory infiltrates, thickening of alveolar septa, vascular congestion, and lung oedema, often associated with acute respiratory distress syndrome (ARDS), respiratory failure, viral sepsis and multiorgan dysfunction [6,7,8]. Due to expression of the angiotensin converting enzyme II receptors on the luminal surface of the gut and colonocytes, SARS-CoV-2 cellular entry is possible and symptoms such as nausea, vomiting, diarrhoea, and abdominal pain are observed in COVID-19 patients. Additionally, COVID-19 has been associated with gut microbial dysbiosis [9].

Clostridium difficile infection (CDI), a serious medical condition of the large intestine, is the leading cause of healthcare-associated diarrhoea in Europe, with a recurrence rate of 15–20% and a mortality rate of 5% [10,11]. The clinical spectrum of CDI ranges from profuse diarrhoea with mucoid, foul smelling, watery stools to severe life-threatening conditions such as pseudomembranous colitis [12].

CDI is usually a consequence of antibiotic exposure, and most cases occur in the elderly population. With the advent of COVID-19, the lack of high-level evidence and rapid viral spread, early management recommendations considered the use of empirical antibiotic treatment, resulting in a large consumption of antimicrobials such as azithromycin [13].

CDI is common in hospitals and is increasingly recognized by experts as a community problem. In addition to its impact on individual patients, CDI accounts for a substantial drain on healthcare resources and costs, however, in many countries, such as Romania, it remains under-recognized by healthcare policymakers, hospital managers, healthcare professionals and the general public [14].

It is difficult to estimate how common CDI is across Europe due to the absence of standardized national surveillance strategies. Reported CDI incidence rates vary widely, which in turn reflects variations in how cases are diagnosed, recorded, and reported. Before the beginning of the COVID-19 pandemic, the incidence of CDI was rising in some European countries and in the United States of America [15]. Moreover, in recent years, there have been outbreaks of particularly severe CDI associated with increased mortality, largely attributed to the spread of a specific type of Clostridium difficile, known in Europe as ribotype 027 [16,17,18,19]. With the identification of the epidemic 027 ribotype, there has been an ongoing debate regarding whether this genetic cluster of *C. difficile* is more virulent than non-epidemic ribotypes, but despite this, it is important to maintain focus on CDI in general rather than the type [20].

According to current reports, the increased focus on hand hygiene, environmental cleaning, patient isolation, and use of personal protective equipment (PPE) during 2020, may have resulted in decreases in healthcare associated infections of CDI during 2020 compared to 2019 [21,22], but taking into consideration the large usage of antibiotics during the current pandemic and the overlapping gastrointestinal symptoms of COVID-19, a renewed attention to CDI is still mandatory.

## 2. Materials and Methods

We conducted an observational, retrospective study on 2065 patients admitted for COVID-19 in the Infectious Diseases Clinic of “Victor Babes” Hospital, Timisoara, from 1 September 2020 until 30 April 2021. During the study period, 2065 COVID-19 patients were admitted. Among them, 109 patients presented, upon admission, respiratory clinical features such as dyspnoea and dry cough, along with gastrointestinal symptoms such as watery stools, emesis, and diffuse abdominal pain. Out of 109 patients with combined symptomatology, 40 patients tested positive for CDI. The remaining patients with dual symptomatology (*n* = 69) formed the control group.

Our main objective was to assess the incidence, clinical characteristics, and outcomes of COVID-19 patients with CDI. In addition, we evaluated risk factors associated with the occurrence of CDI in COVID-19 patients. The study was approved by the Ethics Committee of the hospital, nr. 6631.

Cases were defined as COVID-19 patients (the nucleic acid of SARS-CoV-2 was detected by real-time reverse transcriptase polymerase chain reaction from nasopharyngeal and oropharyngeal swabs) with microbiological evidence of CDI (A/B positive toxins).

Control cases were defined as COVID-19 patients with gastrointestinal symptoms and absent microbiological evidence of CDI.

Demographic, epidemiological, and clinical data (COVID-19 onset and clinical characteristics, medication administered for COVID-19, antimicrobial treatments prescribed before diagnosis of COVID-19, laboratory data, CDI onset and characteristic, and patient’s outcome) were collected. All cases were followed up to 30 days from their hospital discharge to assess the recurrence of CDI and the mortality at 30 days from the hospital discharge.

COVID-19 case confirmation was obtained using the CFX96 Real-Time PCR Systems (Bio-Rad, Hercules, CA, USA). The viral RNA was extracted with the NIMBUS extractor, using the STARMag 96X4 Universal Cartridge Kit (Seegene, Seoul, Korea) and amplified with the Allplex 2019-nCoV (Seegene, Seoul, Korea) kit.

The stool samples were collected in sterile recipients. The etiology was confirmed by the VIDAS^®®^ *C. difficile* Toxin A&B (bioMérieux, Marcy-l’Étoile, France) test, an enzyme linked fluorescent assay (ELFA) that detects toxins A and B in fresh stool samples.

Due to high costs of processing in Romania, ribotyping was performed only in six patients selected by computer randomization. GeneXpert^®®^ (Cepheid, Sunnyvale, CA, USA) C. *difficile* assay polymerase chain reaction allowed distinction between toxin B and binary toxin, as well as the presumptive detection of strain 027/NAP1/BI.

Data analysis was performed using the Statistical Package for Social Sciences v.25 (IBM SPSS Statistics, Chicago, IL, USA). Quantitative variables were tested for normal distribution and compared by means of a paired *t*-test. Qualitative differences between groups were assessed by use of Fisher’s exact test. The precision of odd ratio (OR) was determined by calculating a 95% confidence interval. A *p* value less than 0.05 was considered statistically significant. Variables from the univariate analysis were considered for inclusion in multivariate logistic regressions analysis if *p* value was less than 0.05.

## 3. Results

The demographic and epidemiological data, the clinical characteristics, comorbidities, and outcome of the 40 COVID-19 patients with CDI, and of the 69 controls included in the study, are presented in Table 1. The CDI characteristics, severity, management, and 30 days follow-up of the study group are shown in Table 2.

The mean age of the 40 patients with COVID-19 and CDI was 61 years, ranging between 1 and 91 years. Among them, 62.5%, were female (see Table 1). The mean adjusted Charlson co-morbidity index (CCI) at admission was 6.13. Elevated inflammatory markers and abnormal laboratory findings were observed in all study group patients.

80% of the study group (32/40), presented a hospital onset of CDI with more than 48 h from first symptoms prior hospital admission. On the other hand, 8/40 patients with COVID-19 and CDI, previously in contact with a healthcare facility, presented symptoms onset less than 48 h from hospital admission, thus a community onset (Table 2).

From the study, 38/40 patients were diagnosed with a first episode of CDI, whereas in 5% of the group population a recurrence of CDI was observed. In 85% of the cases (34/40), the diarrhoea onset and the CDI diagnosis followed the COVID-19 diagnosis.

Regarding CDI severity, 20% of the study group patients, developed a mild form of enterocolitis (absent fever, and absent signs of ileus, peritonitis, pseudomembranous colitis, or increased WBC count). Of the COVID-19 patients, 35% with CDI developed a severe form of enterocolitis. Of the study group patients, 45% suffered from complications (admission in the intensive care unit, sepsis, toxic megacolon, death).

Overall, the mean length of the in-hospital stay was 36 days, ranging between 1 and 58 days.

As presented in Table 1, 67.5% of the study group population had a personal history of cardiovascular diseases and 45% had diabetes. Regarding risk factors for CDI, in the two months period prior hospital admission, 75%, 35% and 52.5% CDI patients received antibiotics, proton pump inhibitors and steroid treatment with dexamethasone or methylprednisolone.

Regarding COVID-19 severity, 5/40 patients presented an uncomplicated form of the disease with no evidence of viral pneumonia or hypoxia and 37.5% were admitted with clinical signs of mild COVID-19 pneumonia (fever, dry cough, dyspnoea and SpO2 > 90% on room air). In the study group, 50% of patients presented signs of severe COVID-19 pneumonia (ground-glass and crazy paving lesions affecting more than 50% of the pulmonary parenchyma, severe dyspnoea, SpO2 < 90% on room air and increased inflammatory markers).

As medication administered for COVID-19 during hospital stay, 75% of the patients received LMWH and 52.5% received PPI and steroids. Additionally, 35% of the COVID-19 patients with CDI were treated with broad-spectrum antibiotics (Table 1). The most common antimicrobial class was macrolides. The most common antibiotic prescribed for outpatient treatment of COVID-19 was azithromycin. Outpatient antibiotic courses varied from 5 to 15 days.

Regarding outcomes, 11/40 (27.5%) patients fully recovered and were discharged without complications, 18/40 developed complications upon discharge, and 9/40 (22.5%) patients died in hospital. CDI was the main cause of death in two of these patients, while septic shock was considered the main cause of death in four patients, followed by respiratory failure in two patients and heart failure in one patient. Out of the COVID-19 patients, 80% with CDI were discharged at home and 26 patients were followed up to 30 days from the hospital discharge. For the remaining patients, there are no available data.

Ribotyping was performed by randomization in six patients. The age group, clinical form of CDI and complications are presented in Table 3.

A survival rate of 77.5% was observed for the study group.

Cases and controls were different for previous hospitalizations in the two months before the current admission (*p* = 0.004). The proportion of cases who received broad-spectrum antibiotics during hospital stay was higher among controls (*p* = 0.06).

Logistic regression analysis identified the administration of antibiotics during the hospital stay (OR: 6.7 (95% CI: 2.3–13.20), *p* = 0.004) as independent risk factors associated with CDI in COVID-19 patients (Table 4).

## 4. Discussion

During this COVID-19 pandemic, our hospital experienced overcrowding, but due to the exceptional epidemiological situation, our institution induced reinforcement of all infection control measures and cleaning regiments. Strict isolation measures for infected patients were taken, in addition to limited next of kin visits and patient movement. All healthcare personal used PPE and patients with COVID-19 and CDI were isolated in single rooms or rooms intendent for a maximum two patients. Implementation of these measures have indirectly limited the nosocomial spread of Clostridium difficile, as supported by our results that show a decrease in the incidence density of nosocomial CDI during the period with the maximum incidence of COVID-19. A prospective surveillance study of CDI, conducted by Laza et al. in 2015, identified an incidence of CDI in Victor Babes Hospital, of 20.57/15.70 to 1000 discharged patients in 2013/2014 [24]. An increase in healthcare associated CDI-cases admitted in our hospital is also reported by Marinescu et al. in 2019, after conducting a one-year observational study [25].

Infection prevention and control strategies extended to our hospitalized COVID-19 patients could have limited the transmission from asymptomatic CDI patients who represent an important source [26,27,28], despite this group transmitting less effectively [29]. In addition, limitation of transfers to perform additional tests or elective surgical procedures has reduced the risk of introducing *C. difficile* into the hospital from the community.

Paradoxically, in the management of COVID-19, a viral infection caused by SARS-CoV-2, there was an overuse of antibiotics without clearly defined antimicrobial stewardship guidelines [30,31]. In our study, an extremely high percentage of COVID-19 patients received broad-spectrum of antibiotics prior to and during their hospital stay. Similar percentages have been reported by Sehgal et al. and Khanna et al. in the first half of 2021 [32,33]. Azithromycin was the most frequently prescribed antibiotic for outpatient treatment of COVID-19 as empirical coverage for possible superinfection of the respiratory tract and thus, independently associated with the risk of developing CDI.

The percentage of COVID-19 patients with mild forms of CDI (20%) was lower compared to 60.5% reported by Guido Granata et al. [23]. This can be explained by delayed CDI diagnosis due to the misleading interpretation of gastrointestinal symptoms in COVID-19 patients [34] and highly transmissible strains such as ribotype 027, generally considered for the last decade, to be associated with toxic megacolon, sepsis or death caused by CDI [35]. Nine out of 40 patients died in the hospital and CDI was the main course in two of these patients, one with ribotype 027. Concomitant CDI and COVID-19 can lead to poor outcomes, but the mortality rate we encountered is lower than the rate of 44% previously reported by Sandhu et al. in 2020 [36]. Overall, our study reported a worse outcome for COVID-19 patients without CDI in comparison with CDI COVID-19 patients. The percentage of CDI COVID-19 patients developing complications at discharge was statistically higher than the control group. Our study identified that 65% of the patients had a history of hospitalization up to two months prior to the CDI episode and had longer in-hospital stays than control patients. These finding support the statement that even during COVID-19, in-hospital stay and medication such as PPI, antibiotics or steroids increase the risk of developing CDI.

## 5. Conclusions

During COVID-19, patients who received empirical antibiotics, had a recent history of healthcare exposure or are known with comorbidities such as cardiovascular disease or diabetes, are prone to CDI.

SARS-CoV-2 infection may alter the onset and the clinical course of CDI. However, in the face of the COVID-19 pandemic and the extensive use of broad-spectrum antibiotics with no clinical benefit, clinicians should remain aware of possible CDI and SARS-CoV co-infection. We also underline the importance of infection prevention and rational antimicrobial stewardship guidelines in the management of COVID-19 patients.

A serious limitation of our study was the small number of cases in which PCR ribotyping was performed. Due to high processing costs, in Romania, PCR ribotyping is not commonly performed.

## Figures and Tables

**Table 1:** Demographic data, clinical features, comorbidities, and outcome of the 40 patients with COVID-19 and *C. difficile* infection (CDI) and 69 COVID-19 controls included in the study.

	CDI Patients (*n* = 40)		Control COVID-19 Patients (*n* = 69)	Fisher’s Test (Paired *t* Test for CCI)	Odds Ratio (95% CI)
Female gender		25 (62.5%)	40 (60%)		
Male gender Age (years) Comorbidities		15 (37.5%)	29 (40%)		
		61.22 ± 18.44	54.22 ± 16.22		
	
No comorbidities		2 (5%)	19 (27.5%)	*p* = 0.02	
Cardiovascular disease		27 (67.5%)	20 (29%)	*p* = 0.01	
Heart failure		11 (27.5%)	5 (7.245%)	*p* = 0.02	
Diabetes		18 (45%)	25 (36.23%)	*p* = 0.5	2.3 (1.15–4.46)
Renal failure		5 (12.5%)	5 (7.245%)	*p* = 0.4	
Neurological disease		12 (30%)	6 (8.69%)	*p* = 0.02	
Vasculitis		5 (12.5%)	2 (2.89%)	*p* = 0.08	
Solid cancer		6 (15%)	4 (5.79%)	*p* = 0.1	
Transplant, immunodeficiency, immunosuppression		4 (10%)	0	*p* = 0.06	
Mean age adjusted CCI at admission		6.13	5.59	*p* = 0.02	
Hospitalization in the previous two months		26 (65%)	15 (21.73%)	*p* = 0.004	2.99 (1.41–6.30)
Transferred to the hospital from a LTHCF		14 (35%)	26 (37.6%)	*p* = 0.8	1.6 (0.76–3.6)
Proton pump inhibitors in the previous two months		14 (35%)	39 (56.52%)	*p* = 0.19	0.61 (0.30–1.2)
Antibiotics in the previous two months		30 (75%)	32 (46.37%)	*p* = 0.1	1.8 (0.87–3.74)
Steroids in the previous two months *		21 (52.5%)	20 (29%)	*p* = 0.1	
COVID-19 severity					
Asymptomatic		5 (12.5%)	10 (14.49%)	*p* = 0.7	
Mild pneumonia		15 (37.5%)	22 (31.88%)	*p* = 0.6	
Severe pneumonia		25 (62.5%)	37 (53.62%)	*p* = 0.6	0.9 (0.47–1.8)
Medication for COVID-19 during the hospital stay					
Remdesivir		19 (47.5%)	37 (53.62%)	*p* = 0.7	
Favipiravir		19 (47.5%)	32 (46.37%)	*p* = 0.9	
Biologics		19 (47.5%)	30 (43.47%)	*p* = 0.8	
LMWH		30 (75%)	52 (75.36%)	*p* = 0.9	
Steroids		21 (52.5%)	48 (69.56%)	*p* = 0.3	
Proton pump inhibitors		21 (52.5%)	48 (69.56%)	*p* = 0.05	
Antibiotics		14 (35%)	47 (68.11%)	*p* = 0.06	
Patient outcome					
Recovered without complications **		11(27.5%)	35 (51%)	*p* = 0.1	0.54 (0.24–1.18)
Recovered with complications		20 (50%)	13 (19%)	*p* = 0.001	2.6 (1.19–5.90)
Deceased		9 (22.5%)	21 (30%)	*p* = 0.4	0.73 (0.30-1.7)
Total length of in hospital stay (days)		36 (range 1–58 days)	28 (range 4–48 days)	*p* = 0.01	

Legend: CCI: Charlson Co-morbidity index, LTHCF: long-term healthcare facility, LMWH: Low molecular wight heparin, * dexamethasone or methylprednisolone, ** discharged with muscle weakness, pressure ulcers or chronic heart decompensation.

**Table 2:** CDI characteristics, severity, management, outcome, and 30 days follow-up of the 40 COVID-19 patients with CDI.

	CDI Patients (*n* = 40)	CDI Patients (Percent %)
Hospital onset CDI	32	80
Community-onset, healthcare-associated CDI	8	20
Recurrence of CDI	2	5
Diarrhoea onset before the COVID-19 diagnosis	6	15
Diarrhoea onset after the COVID-19 diagnosis	34	85
CDI severity at diagnosis		
Mild Severe Complicated	8 14 18	20 35 45
Administered anti-CD antimicrobial treatment		
Vancomycin Vancomycin and Metronidazole Metronidazole Metronidazole and Rifaximin Vancomycin and Rifaximin	14 4 5 4 13	35 10 12.5 10 32.5
Follow up at 30 days from the discharge		
Deceased before the discharge Follow up after discharge not available	9 2	22.5 5
Patients followed-up at 30 days from the discharge	26	
Recovered at home, no subsequent rCDI Readmission in hospital Deceased, CDI-related Deceased, not CDI-related	16 1 2 7	40 2.5 5 17.5

Legend: rCDI: recurrent CDI episode (after at least two days from the resolution of the diarrhoea and after the end of the antimicrobial treatment of the previous CDI episode [23]).

**Table 3:** CDI ribotype, clinical features, treatment, and outcome.

		CDI Patients (*n* = 6)
Age group	<40 years old	2
	40–59 years old	3
	60–70 years old	1
Ribotype	027	6
Clinical form of CDI	mild	-
	severe	3
	complicated	3
Treatment	vancomycin	2
	vancomycin and rifaximin	4
Outcome	deceased, CDI-related	1
	recovered at home, no subsequent rCDI	5

**Table 4:** Factors associated with likelihood of CDI during COVID-19 infection. Logistic regression analysis.

	CDI Patients (*n* = 40)	Control COVID-19 Patients (*n* = 69)	Univariate Analysis		Multivariate Analysis	
			*p*-Value	Odds Ratio (95% CI)	*p*-Value	Odds Ratio (95% CI)
Comorbidities						
Cardiovascular disease	27 (67.5%)	20 (29%)	*p* = 0.01	2.32 (1.15–4.67)	*p* = 0.9	
Transplant, immunodeficiency, immunosuppression	4 (10%)	0	*p* = 0.06	15.44 (0.81–294.30)	*p* = 0.1	
Hospitalization in the previous two months	26 (65%)	15 (21.73%)	*p* = 0.004	2.99 (1.41–6.30)	*p* = 0.07	5.6 (2.3–10.9)
Transferred to the hospital from a LTHCF	14 (35%)	26 (37.6%)	*p* = 0.8	1.6 (0.76–3.6)	*p* = 0.015	8.4 (2.3–30.5)
Proton pump inhibitors in the previous two months	14 (35%)	39 (56.52%)	*p* = 0.19	0.61 (0.30–1.2)	*p* = 0.09	
Antibiotics in the previous two months	30 (75%)	32 (46.37%)	*p* = 0.1	1.8 (0.87–3.74)	*p* = 0.1	
Steroids in the previous two months	21 (52.5%)	20 (29%)	*p* = 0.1	1.8 (0.87–3.74)	*p* = 0.1	
Antibiotics	14 (35%)	47 (68.11%)	*p* = 0.06	0.51 (0.25–1.04)	*p* = 0.004	6.7 (2.3–13.20)

## Data Availability

The data presented in this study is openly available online: http://dx.doi.org/10.17632/8pbkt9t64x.1.
